# Spatial Patterns of Whole Brain Grey and White Matter Injury in Patients with Occult Spastic Diplegic Cerebral Palsy

**DOI:** 10.1371/journal.pone.0100451

**Published:** 2014-06-25

**Authors:** Xuetao Mu, Binbin Nie, Hong Wang, Shaofeng Duan, Zan Zhang, Guanghui Dai, Qiaozhi Ma, Baoci Shan, Lin Ma

**Affiliations:** 1 Department of Radiology, Chinese PLA General Hospital, Beijing, China; 2 Department of MRI, The General Hospital of Chinese People's Armed Police Forces, Beijing, China; 3 Key Laboratory of Nuclear Analysis Techniques, Beijing Engineering Research Center of Radiographic Techniques and Equipment, Institute of High Energy Physics, Chinese Academy of Sciences, Beijing, China; 4 Neurosurgical Institute, The General Hospital of Chinese People's Armed Police Forces, Beijing, China; Institute of Psychology, Chinese Academy of Sciences, China

## Abstract

Spastic diplegic cerebral palsy(SDCP)is a common type of cerebral palsy (CP), which presents as a group of motor-impairment syndromes. Previous conventional MRI studies have reported abnormal structural changes in SDCP, such as periventricular leucomalacia. However, there are roughly 27.8% SDCP patients presenting normal appearance in conventional MRI, which were considered as occult SDCP. In this study, sixteen patients with occult SDCP and 16 age- and sex-matched healthy control subjects were collected and the data were acquired on a 3T MR system. We applied voxel-based morphometry (VBM) and tract-based spatial statistics (TBSS) analysis to investigate whole brain grey and white matter injury in occult SDCP. By using VBM method, the grey matter volume reduction was revealed in the bilateral basal ganglia regions, thalamus, insula, and left cerebral peduncle, whereas the white matter atrophy was found to be located in the posterior part of corpus callosum and right posterior corona radiata in the occult SDCP patients. By using TBSS, reduced fractional anisotropy (FA) values were detected in multiple white matter regions, including bilateral white matter tracts in prefrontal lobe, temporal lobe, internal and external capsule, corpus callosum, cingulum, thalamus, brainstem and cerebellum. Additionally, several regions of white matter tracts injury were found to be significantly correlated with motor dysfunction. These results collectively revealed the spatial patterns of whole brain grey and white matter injury in occult SDCP.

## Introduction

Cerebral palsy (CP) is a motor-impairment syndrome resulting from genetic or acquired disorders in the early brain development. About 70 to 90% of CP children presented grey and white matter abnormality on the conventional magnetic resonance imaging (MRI). Although conventional MRI is valuable in the identification of brain injuries, there are still 10–30% CP patients presenting normal appearance in conventional MRI [1], [2]. We take those patients as occult CP. Is the brain really normal or undetectably abnormal in conventional MRI?

Voxel-based morphometry (VBM) as an automated technique are more sensitive than conventional MRI in investigating the structural changes of the whole-brain [3]–[7]. A recent MRI study has employed VBM to detect regional grey matter and white matter volume abnormalities in CP [6]. However, previous studies mainly focused on CP patients who exhibited abnormal findings in conventional MRI. No attempt has been made to investigate grey and white matter changes in occult CP using this automated methodology. In the present study, we applied VBM to occult CP to explore the possible grey and white matter injury.

With the advancement of diffusion tensor imaging (DTI) techniques, it has been made possible to non-invasively examine the whole brain white matter integrity in vivo. For the analysis of DTI data, fractional anisotropy (FA) is considered the most important diffusion metrics which reflects the degree of directionality of cellular structures within the fiber tracts by measuring anisotropic water diffusion [8], [9]. There have been several studies directed at exploring the white matter integrity in CP using DTI method [10]–[12]. Unlike the previous region of interest (ROI) analysis method, we employed a new voxel-wise DTI analysis method, which is called tract-based spatial statistics (TBSS), to reveal the whole brain white matter injury in occult CP [13].

Spastic diplegic cerebral palsy(SDCP)is a common type of CP. Previous conventional MRI studies have reported that normal appearance rate of SDCP can be as high as 27.8% [2], [14]. Therefore, we selected patients with this type of CP as the subjects. We hypothesized that the occult SDCP might show grey and white matter injury in specific regions depending on the pathology of the disease. We had two objectives in mind. The first objective was to detect grey and white matter changes in occult SDCP using the whole brain VBM and TBSS technique. The second one was to investigate the relationship between the structural changes and motor dysfunction in occult SDCP.

## Materials and Methods

### Subjects

From October 2008 to August 2013, a total of 121 SDCP patients aged between 2 and 14 years were included. Conventional MRI examinations were performed in all patients. Of the 121 patients, 18 patients fulfilled the diagnostic criteria of occult SDCP. Neurological examinations were performed by two pediatric neurologists (DGH and ZZ) to corroborate the findings. The motor dysfunction scale was evaluated using the gross motor function classification system (GMFCS). MR images were interpreted and consensus about the interpretation was reached among 3 radiologists (MXT, WH, and MQZ). After the study was approved by General Hospital of Armed Police review board, written informed consent was obtained from all the parents according to the Declaration of Helsinki.

Inclusion criteria of the occult SDCP in the study were as follows: 1) the age ranged from 2 to 14 years; 2) the patients must meet the diagnostic criteria of CP. Firstly, clinical diagnosis of a non-progressive motor impairment was made due to a presumably early insult to the developing brain; secondly, The patients may have one or more of the following symptoms: cognitive disabilities, language impairment, seizure, sensory loss, and/or musculoskeletal abnormalities; finally, objective evidence of neuromotor impairment, as manifested by abnormal muscle tone, strength, posture, reflexes, and/or motor skills, must be confirmed on physical examinations; 3) the patients must have spastic diplegia symptoms, in which the lower limbs were more severely affected than the upper limbs. Spasticity refers to a velocity-dependent increase in the muscle tone (resistance to stretch). Diplegia means bilateral involvement; 4) the brain showed normal-appearing in conventional MRI, including T1 and T2 weighted imaging [14].

Exclusion criteria were as follows: 1) static movement disorders with an onset after the age of 3, as well as progressive movement disorders with varying degrees of severity or psychomotor retardation attributable to progressive tumor, metabolic, degenerative, or genetic causes; the presence of sensory deficits, ataxia, muscle atrophy, involuntary movements occurring in the development; movement disorders caused by motor neuron disease; 2) Data scan failure due to movement in the MRI examination.

Eighteen patients fulfilled the above mentioned criteria and were included. After evaluation of the high resolution 3D structural images, 2 patients were excluded due to poor image quality. Altogether, 16 patients participated in our study and 16 healthy children were enrolled in the normal control group. The inclusion criteria of the control group were as follows: full-term natural birth, no history of ischemia, anoxia, or genetic disorder, completely normal intelligence and motor function, and normal appearance on conventional MRI. The age and gender matched in the two groups (Table 1).

**Table 1:** Demographic and clinical data of occult SDCP patients and normal controls.

Group(n)	Sex (M/F)	Age(years) (mean±SD)	Age range (years)	Clinical factors(n)	GMFC (n)
Occult SDCP(16)	9/7	8.31±4.00	2–14	premature birth(4)	I(8)
				low birth weight(3)	II(4)
				perinatal asphyxia(9)	III(4)
NC(16)	10/6	7.56±3.33	2–14	_	_
P value	0.4386	0.5682	_	_	_

SDCP, spastic diplegic cerebral palsy; NC, normal control; M, male; F, female; SD, standard deviation; GMFCS, gross motor function classification system.

### Image acquisition

A 3.0 T MR scanner (TRIO TIM, Siemens Medical Systems, Germany) with a SENSE 8-channel head coil was used. Conventional MRI study with standard imaging sequences preceded the VBM research protocol.

Routine clinical pulse sequences were obtained in all subjects, including axial T1-weighted fast low-angle shot sequence [repetition time (TR)/echo time (TE) =  1900/2.5 ms, slice thickness = 5 mm, intersection gap = 1.5 mm, slice number = 19, field of view (FOV) = 220×220 mm, band width (BW) = 269 Hz, flip angle (FA) = 70°], sagittal and axial T2-weighted turbo spin echo sequence (TR/TE = 4000/87 ms, slice thickness = 5 mm, intersection gap = 1.5 mm, slice number = 19, FOV = 220×220 mm, BW = 363 Hz, FA = 140°), and axial fluid-attenuated inversion recovery sequence (TR/TE = 8000/113 ms, inversion time 2000 ms, slice thickness = 5 mm, intersection gap = 1.5 mm, slice number = 19, FOV = 220×220 mm, BW =  269 Hz, FA = 120°).

High resolution 3D structural images were acquired by using magnetization prepared rapid gradient echo sequence parallel to the corpus callosum (TR/TE = 1900/2.58 ms, slice thickness = 0.9 mm, intersection gap = 0 mm, number of slices = 176, FOV =  240 mm×204 mm, BW = 170 Hz, FA = 15°, scan time = 3.38 minute, voxel size = 0.9 mm×0.9 mm×0.9 mm), covering the entire brain.

DTI were acquired by using spin-echo diffusion-weighted echo-planar imaging sequence parallel to the anterior -posterior commissure line (TR/TE = 4400/93 ms, slice thickness = 4 mm, intersection gap = 0 mm, number of slices = 32, FOV = 220 mm×220 mm, matrix  = 128×128, voxel size  = 1.7 mm×1.7 mm×4.0 mm, MRI images were obtained from 20 non-collinear directions with a b value of 1000 s/mm^2^, averages times  = 4, scan time = 6.24 minute).

### Image analysis

The processing and data analysis were performed using the VBM2, an optimized VBM method [15], [16], which is an extension toolbox of the SPM2 (Welcome Department of Cognitive Neurology, London, UK). A study-specific whole brain T1-weighted template and prior images of grey matter, white matter, and cerebrospinal fluid (CSF) were created based on the Montreal Neurological Institute (MNI) template in SPM2. Each participant's original image was spatially normalized based on the customized template and subsequently segmented into GM, WM and CSF based on the customized priors. All the segmented images were resliced by 1.0×1.0×1.0 mm^3^ voxels. Furthermore, voxel values in segmented images of grey and white matter were multiplied by the Jacobian determinants to preserve within-voxel volumes that may have been altered during non-linear normalization. This procedure yielded ‘modulated’ images, which were used for the group comparison of grey matter volume (GMV) and white matter volume (WMV). Eventually, all the GMV and WMV images were smoothed by a Gaussian kernel of 8 mm full width at half-maximum (FWHM).

The preprocessed images were analyzed within SPM2 based on the framework of the general linear model [17]. In order to identify the difference in GMV and WMV between SDCP patients and the normal controls, two-sample t-test [18] was performed using SPM2. Brain regions with significant GMV and WMV changes in patients were yielded based on a voxel-level height threshold of p<0.001(uncorrected) and a cluster-extent threshold of 50 voxels. Then the voxel-wise correlation between GMV, WMV and GMFCS levels was performed, with P<0.05 as statistically significant threshold.

Voxel wise statistical analysis of the FA data was carried out using DTI-studio [19] and TBSS [13] which is a part of FSL [20]. FA images were created by fitting a tensor model to the raw diffusion data using DTI-studio. All subjects' FA data were then aligned into a common space using the nonlinear registration tool FNIRT [21], [22], which utilizes a b-spline representation of the registration warp field [23]. The mean FA image was created and thinned to create a mean FA skeleton which represents the centers of all tracts common to the group. A threshold of FA > 0.2 was applied to exclude unmajor fiber tracts. Each subject's aligned FA data was then projected onto this skeleton and the resultant data fed into voxel wise cross-subject statistics. Statistical analysis was performed with TFCE method [24],which was achieved by using ‘randomise’ command with ‘-tfce’ option in FSL [25]. The ‘randomise’ is a permutation test, and 5000 times permutation were performed to fit the group model. Regions with significant FA changes were yielded based on a voxel-level height threshold of P<0.001 (uncorrected). The ensuing voxel-wise correlation between FA and GMFCS levels was performed, with P<0.05 as statistically significant threshold.

## Results

### Demographic and clinical data of the study groups

Descriptive characteristics and neuropsychological scores were shown in Table 1. No significant differences in age and gender were noted between the patients with occult SDCP and normal controls (p>0.05). Of the occult SDCP patients, 4 patients were born prematurely, 3 patients were with low birth weight, and 9 patients had history of perinatal asphyxia. By GMFCS, 12 patients had a mild degree of motor dysfunction (Level I = 8, Level II = 4), and 4 patients had moderate degree of motor dysfunction (Level III = 4).

### Grey matter and white matter volume loss in occult SDCP using VBM method

**Figure 1:**
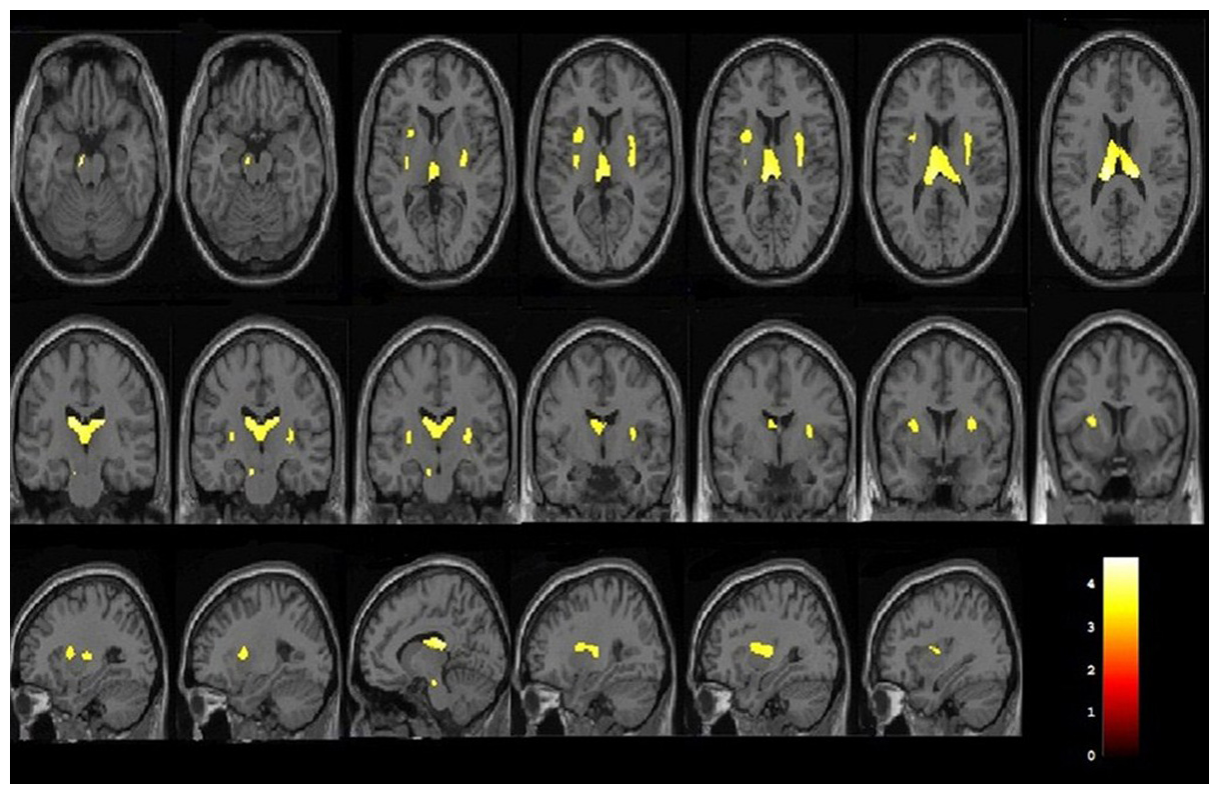
Grey matter volume differences between occult SDCP patients and normal controls by VBM analysis. Occult SDCP patients showed significantly decreased grey matter volume in bilateral lentiform nucleus, claustrum, thalamus, insula, and left cerebral peduncle. Reader's left is subject's left.

**Figure 2:**
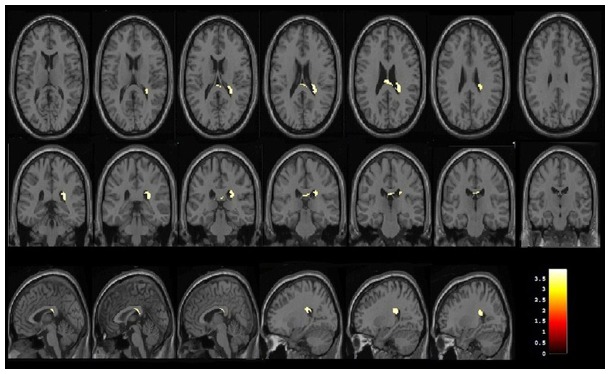
White matter volume differences between occult SDCP patients and normal controls by VBM analysis. Occult SDCP patients showed significantly decreased white matter volume in posterior part of corpus callosum and right posterior corona radiata. Reader's left is subject's left.

Grey matter volume reduction was found in the bilateral basal ganglia regions (lentiform nucleus and claustrum), thalamus, insula, and left cerebral peduncle in the occult SDCP patients, compared with the normal controls (Figure 1 and Table 2). In contrast with the normal controls, white matter volume reduction was located in the posterior part of corpus callosum and right posterior corona radiata in the occult SDCP patients (Figure 2 and Table 2). There was no significant grey or white matter volume increase in occult SDCP patients compared with the normal controls in this study.

**Table 2:** Areas of grey and white matter volume loss in occult SDCP patients compared with NC.

Anatomic area	Talairach coordinate			Cluster size	Z scores	*P* value
	x	y	z			
Grey matter						
L cerebral peduncle	−7	−15	−21	136	3.67	0.000
R-L thalamus	−13	−25	19	6437	3.95	0.000
	18	−25	20		3.91	0.000
	−2	−26	11		3.73	0.000
R lentiform nucleus/claustrum/insula	31	−14	10	1538	3.48	0.000
	30	2	13		3.37	0.000
L lentiform nucleus	−29	−14	9	302	3.34	0.000
L lentiform nucleus/claustrum/insula	−26	8	10	765	3.39	0.000
White matter						
R posterior corona radiata	25	−32	21	1167	3.47	0.000
corpus callosum	4	−23	23	410	3.4	0.000
	0	−28	18		3.2	0.001

NC, normal control; R, right; L, left.

### White matter injury in occult SDCP using TBSS method

**Figure 3:**
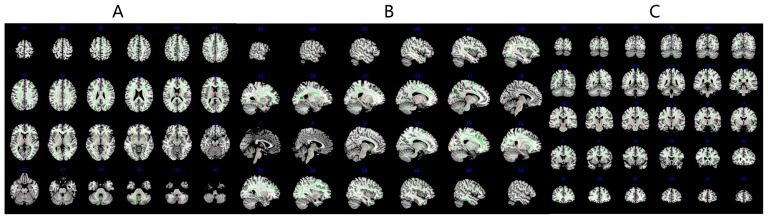
TBSS results of FA values differences between occult SDCP patients and normal controls groups. Green is the mean white matter skeleton of all subjects; red is the regions with reduced FA in occult SDCP patients. TBSS results for increased FA in occult SDCP patients did not show any statistically significant differences at P<0.001 (uncorrected). (A) axial position; (B) sagittal position; (C) coronal position. Reader's left is subject's left.

The differences of FA values between patients with occult SDCP and healthy controls were revealed by TBSS. Compared with the healthy controls, occult SDCP patients showed significantly reduced FA in multiple white matter regions, including bilateral white matter tracts in prefrontal lobe, temporal lobe, internal and external capsule, corpus callosum, cingulum, thalamus, brainstem and cerebellum (Figure 3 A–C). There were no significantly increased FA regions in the white matter in occult SDCP patients compared with the normal controls in this study.

### Correlation of the severity of white matter injury with GMFCS levels

**Figure 4:**
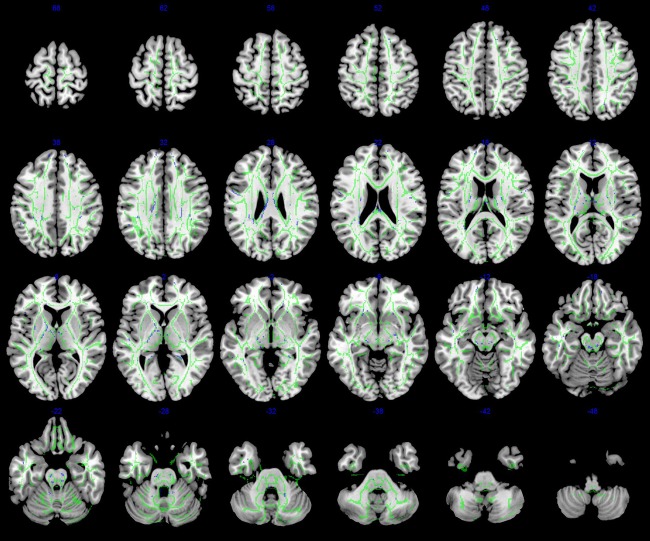
Voxel-wise correlation analysis between FA values and GMFCS levels on TBSS. Green is the mean white matter skeleton of all subjects; Areas in blue represent regions showing significant negative correlation between FA values and GMFCS levels. Reader's left is subject's left.

Grey and white matter volume reduction did not show significant correlation with motor dysfunction. FA values within bilateral white matter tracts of prefrontal lobe, thalamus, internal capsule, corpus callosum and brainstem showed a negative correlation with GMFCS levels (P<0.05) (Figure 4)

## Discussion

Patients with SDCP whose brain abnormalities are unobservable on conventional MRI are termed “occult SDCP” in this study [1]. However, subtle structural injury may exist in these patients, which, supposedly, is closely related to motor dysfunction. We combined two highly sensitive techniques including VBM and TBSS to reveal the grey and white matter abnormalities in occult SDCP in the hope of offering a clearer understanding of the disease. By using VBM method, the occult SDCP showed grey matter reduction in multiple brain regions mostly concentrated in basal ganglia, thalamus, insula, while white matter reduction tended to be found in posterior corpus callosum and the right corona radiata. As shown by TBSS, occult SDCP exhibited significantly reduced FA values in several white matter tracts, some of which including prefrontal lobe, thalamus, internal capsule, corpus callosum and brainstem showed significant negative correlation with motor dysfunction scores.

Previous studies have shown grey matter volume reductions in the CP patients, which suggested neuronal degeneration and damage [26]–[31]. As the basal ganglia regions are very sensitive to the prenatal hypoxic-ischemic insult, these regions are vulnerable to injuries and degeneration, as was confirmed by recent studies [6], [11]. The thalamus atrophy is also an important finding in occult SDCP patients. Recent neuropathological studies indicated the damage of thalamus in CP due to diffuse neuronal loss and gliosis [32]. In previous CP studies, several quantitative volumetric MRI studies demonstrated the decreased volume of thalamus in CP patients [27], [33], [34]. Furthermore, DTI measurements of the thalamus also revealed a significant reduction of FA value in bilateral thalamus [10], [11]. In addition, insula was also found to be involved. As insula is adjacent to the basal ganglia regions where connections exist, its volume reduction might result from neuronal degeneration occasioned by hypoxic-ischemic insult. Nevertheless, we did not find grey matter atrophy in other regions, such as sensorimotor cortex, frontal, parietal, or occipital lobes, which finding was different from those reported by previous studies. Considering that only occult SDCP was studied, we speculated that the basal ganglia, thalamus and insula were preferential and sensitive regions, which, presumably, are the very early affected sites in SDCP.

By using VBM method, we also found white matter injuries in occult SDCP, which were chiefly located in the posterior corpus callosum and right posterior corona radiata. Previous studies demonstrated that white matter pathology was an early marker of the disease, which mainly presented with gliosis and microscopic cystic changes resulting from hypoxic-ischemic insult [11]. According to previous CP studies, diminished motor cortical connectivity and the corticospinal tract could be the pathophysiological mechanism responsible for motor dysfunction. Because the bilateral motor cortex is connected by the posterior part of corpus callosam, and posterior corona radiata is part of parieto-occipital white matter tract, we speculated that posterior corpus callosum and corona radiata might be the predilection white matter injury site of SDCP.

One of the advantages of DTI over VBM, which is capable of identifying the subtle white matter injury, is that it can provide fuller and more detailed information on white matter microstructure injury based on the diffusion properties. In this study, we compared the group differences in FA values between occult SDCP patients and healthy controls using a novel TBSS method, and found the diffuse white matter injury in multiple brain regions such as prefrontal lobe, temporal lobe, internal and external capsule, corpus callosum, cingulum, thalamus, brainstem and cerebellum. A previous DTI study showed marked reduction of white matter in CP patients mainly in the internal capsule, posterior thalamic radiation, superior corona radiata and commissural fibers [35]. Another study using DTI method showed FA values were significantly lower in posterior white matter tracts and corpus callosum in CP patients [6]. In recent DTI study, CP patients showed significantly decreased FA values in corona radiata, internal capsule, mid-brain, pons, medulla, genu and splenium of corpus callosum, and occipital white matter [36]. In a recent DTI study, significant decrease of FA value was limited to splenium of the corpus callosum, thalamus, and periventricular deep white matter in spastic CP patients [37]. Based on a recent review by using DTI, the most common regions of decreased FA values were in corticospinal tracts and thalamic radiations, indicating disruption of the corticomotor and sensorimotor neural networks in CP [38]. Our results were consistent with those previous studies.

Since the periventricular white matter and basal ganglion are more sensitive to ischemia and are most easily affected regions by hypoxic-ischemic insults, both volume loss (by VBM) and decreased FA (by TBSS) were revealed in those regions. However, decreased FA values without volume loss appeared in more widespread regions including cerebrum and cerebellum. The reason may be the relative insensitivity to ischemic injury in those widespread brain regions, resulting in diffusion abnormality of FA values but with no apparent volume loss.

We further explored the correlation between motor function scales and FA values at every voxel level. The results demonstrated that the severity of white matter injury within prefrontal lobe, thalamus, internal capsule, corpus callosum and brainstem showed significant negative correlation with motor dysfunctions, as measured by GMFCS levels. Previous CP studies demonstrated FA values within corticospinal tracts, posterior corpus callosum and thalamic radiation were significantly correlated with motor dysfunction [6], [39], [40]. Adopting different sampling and analysis methods, we showed the consistent results. Collectively, we speculated that these regions may constitute motor pathway and play important roles in the motor function. These results will provide potential neuroimaging markers for evaluation of motor function in future treatment of SDCP.

The limitation of the present study was that only the occult SDCP was investigated. In the future, comparative analysis of the brain structure changes among normal control, occult SDCP, and SDCP should be further performed, which would be more helpful to reveal the pathological mechanisms of SDCP.

In conclusion, by using the VBM and TBSS method, the occult SDCP patients were found to present with selective grey and white matter injury. And the white matter tracts injury in some regions was found to be significantly correlated with motor function. These findings are expected to offer a deeper insight into the pathophysiological mechanisms of occult SDCP.

## References

[1] Bax M, Tydeman C, Flodmark O (2006) Clinical and MRI correlates of cerebral palsy: the European Cerebral Palsy Study. JAMA 296: 1602–1608. 17018805 10.1001/jama.296.13.1602

[2] Benini R, Dagenais L, Shevell MI, Registre de la Paralysie Cerebrale au Quebec C (2013) Normal imaging in patients with cerebral palsy: what does it tell us? J Pediatr 162: 369–374 e361. 10.1016/j.jpeds.2012.07.044 22944004

[3] Wang Z, Guo X, Qi Z, Yao L, Li K (2010) Whole-brain voxel-based morphometry of white matter in mild cognitive impairment. Eur J Radiol 75: 129–133. 19443157 10.1016/j.ejrad.2009.04.041

[4] Tan L, Fan Q, You C, Wang J, Dong Z, et al (2013) Structural changes in the gray matter of unmedicated patients with obsessive-compulsive disorder: a voxel-based morphometric study. Neurosci Bull 29: 642–648. 23990196 10.1007/s12264-013-1370-7 PMC5561964

[5] Carducci F, Onorati P, Condoluci C, Di Gennaro G, Quarato PP, et al (2013) Whole-brain voxel-based morphometry study of children and adolescents with Down syndrome. Funct Neurol 28: 19–28. 23731912 PMC3812718

[6] Lee JD, Park HJ, Park ES, Oh MK, Park B, et al (2011) Motor pathway injury in patients with periventricular leucomalacia and spastic diplegia. Brain 134: 1199–1210. 21385750 10.1093/brain/awr021

[7] Padovani A, Borroni B, Brambati SM, Agosti C, Broli M, et al (2006) Diffusion tensor imaging and voxel based morphometry study in early progressive supranuclear palsy. J Neurol Neurosurg Psychiatry 77: 457–463. 16306152 10.1136/jnnp.2005.075713 PMC2077489

[8] Pierpaoli C, Basser PJ (1996) Toward a quantitative assessment of diffusion anisotropy. Magn Reson Med 36: 893–906. 8946355 10.1002/mrm.1910360612

[9] Le Bihan D, Mangin JF, Poupon C, Clark CA, Pappata S, et al (2001) Diffusion tensor imaging: concepts and applications. J Magn Reson Imaging 13: 534–546. 11276097 10.1002/jmri.1076

[10] Nagasunder AC, Kinney HC, Bluml S, Tavare CJ, Rosser T, et al (2011) Abnormal microstructure of the atrophic thalamus in preterm survivors with periventricular leukomalacia. AJNR Am J Neuroradiol 32: 185–191. 20930003 10.3174/ajnr.A2243 PMC3281310

[11] Thomas B, Eyssen M, Peeters R, Molenaers G, Van Hecke P, et al (2005) Quantitative diffusion tensor imaging in cerebral palsy due to periventricular white matter injury. Brain 128: 2562–2577. 16049045 10.1093/brain/awh600

[12] Fan GG, Yu B, Quan SM, Sun BH, Guo QY (2006) Potential of diffusion tensor MRI in the assessment of periventricular leukomalacia. Clin Radiol 61: 358–364. 16546466 10.1016/j.crad.2006.01.001

[13] Smith SM, Jenkinson M, Johansen-Berg H, Rueckert D, Nichols TE, et al (2006) Tract-based spatial statistics: voxelwise analysis of multi-subject diffusion data. Neuroimage 31: 1487–1505. 16624579 10.1016/j.neuroimage.2006.02.024

[14] Towsley K, Shevell MI, Dagenais L, Consortium R (2011) Population-based study of neuroimaging findings in children with cerebral palsy. Eur J Paediatr Neurol 15: 29–35. 20869285 10.1016/j.ejpn.2010.07.005

[15] Ashburner J, Friston KJ (2000) Voxel-based morphometry–the methods. Neuroimage 11: 805–821. 10860804 10.1006/nimg.2000.0582

[16] Good CD, Ashburner J, Frackowiak RS (2001) Computational neuroanatomy: new perspectives for neuroradiology. Rev Neurol (Paris) 157: 797–806. 11677400

[17] Friston KJ, Holmes AP, Worsley KJ, Poline JP, Frith CD, et al (1994) Statistical parametric maps in functional imaging: a general linear approach. Human brain mapping 2: 189–210.

[18] Friston KJ, Ashburner JT, Kiebel SJ, Nichols TE, Penny WD (2011) Statistical Parametric Mapping: The Analysis of Functional Brain Images: The Analysis of Functional Brain Images: Academic Press.

[19] Jiang H, van Zijl PC, Kim J, Pearlson GD, Mori S (2006) DtiStudio: resource program for diffusion tensor computation and fiber bundle tracking. Comput Methods Programs Biomed 81: 106–116. 16413083 10.1016/j.cmpb.2005.08.004

[20] Smith SM, Jenkinson M, Woolrich MW, Beckmann CF, Behrens TE, et al (2004) Advances in functional and structural MR image analysis and implementation as FSL. Neuroimage 23 Suppl 1 S208–219. 15501092 10.1016/j.neuroimage.2004.07.051

[21] Andersson JL, Jenkinson M, Smith S, Andersson J (2007) Non-linear optimisation. FMRIB technical report TR07JA1. Oxford (UK): FMRIB Centre.

[22] Andersson JL, Jenkinson M, Smith S (2007) Non-linear registration, aka Spatial normalisation FMRIB technical report TR07JA2. FMRIB Analysis Group of the University of Oxford.

[23] Rueckert D, Sonoda LI, Hayes C, Hill DL, Leach MO, et al (1999) Nonrigid registration using free-form deformations: application to breast MR images. IEEE Trans Med Imaging 18: 712–721. 10534053 10.1109/42.796284

[24] Smith SM, Nichols TE (2009) Threshold-free cluster enhancement: addressing problems of smoothing, threshold dependence and localisation in cluster inference. Neuroimage 44: 83–98. 18501637 10.1016/j.neuroimage.2008.03.061

[25] Nichols TE, Holmes AP (2002) Nonparametric permutation tests for functional neuroimaging: a primer with examples. Hum Brain Mapp 15: 1–25. 11747097 10.1002/hbm.1058 PMC6871862

[26] Inder TE, Huppi PS, Warfield S, Kikinis R, Zientara GP, et al (1999) Periventricular white matter injury in the premature infant is followed by reduced cerebral cortical gray matter volume at term. Ann Neurol 46: 755–760. 10553993 10.1002/1531-8249(199911)46:5<755::aid-ana11>3.0.co;2-0

[27] Inder TE, Warfield SK, Wang H, Huppi PS, Volpe JJ (2005) Abnormal cerebral structure is present at term in premature infants. Pediatrics 115: 286–294. 15687434 10.1542/peds.2004-0326

[28] Northington FJ, Graham EM, Martin LJ (2005) Apoptosis in perinatal hypoxic-ischemic brain injury: how important is it and should it be inhibited? Brain Res Brain Res Rev 50: 244–257. 16216332 10.1016/j.brainresrev.2005.07.003

[29] Pierson CR, Folkerth RD, Billiards SS, Trachtenberg FL, Drinkwater ME, et al (2007) Gray matter injury associated with periventricular leukomalacia in the premature infant. Acta Neuropathol 114: 619–631. 17912538 10.1007/s00401-007-0295-5 PMC2080348

[30] Stone BS, Zhang J, Mack DW, Mori S, Martin LJ, et al (2008) Delayed neural network degeneration after neonatal hypoxia-ischemia. Ann Neurol 64: 535–546. 19067347 10.1002/ana.21517 PMC2605201

[31] Leviton A, Gressens P (2007) Neuronal damage accompanies perinatal white-matter damage. Trends Neurosci 30: 473–478. 17765331 10.1016/j.tins.2007.05.009

[32] Ligam P, Haynes RL, Folkerth RD, Liu L, Yang M, et al (2009) Thalamic damage in periventricular leukomalacia: novel pathologic observations relevant to cognitive deficits in survivors of prematurity. Pediatr Res 65: 524–529. 19127204 10.1203/PDR.0b013e3181998baf PMC2713790

[33] Woodward LJ, Anderson PJ, Austin NC, Howard K, Inder TE (2006) Neonatal MRI to predict neurodevelopmental outcomes in preterm infants. N Engl J Med 355: 685–694. 16914704 10.1056/NEJMoa053792

[34] Thompson DK, Warfield SK, Carlin JB, Pavlovic M, Wang HX, et al (2007) Perinatal risk factors altering regional brain structure in the preterm infant. Brain 130: 667–677. 17008333 10.1093/brain/awl277

[35] Nagae LM, Hoon AH Jr, Stashinko E, Lin D, Zhang W, et al (2007) Diffusion tensor imaging in children with periventricular leukomalacia: variability of injuries to white matter tracts. AJNR Am J Neuroradiol 28: 1213–1222. 17698519 10.3174/ajnr.A0534 PMC7977654

[36] Rai Y, Chaturvedi S, Paliwal VK, Goyal P, Chourasia A, et al (2013) DTI correlates of cognition in term children with spastic diplegic cerebral palsy. Eur J Paediatr Neurol 17: 294–301. 23246381 10.1016/j.ejpn.2012.11.005

[37] Yoshida S, Faria AV, Oishi K, Kanda T, Yamori Y, et al (2013) Anatomical characterization of athetotic and spastic cerebral palsy using an atlas-based analysis. J Magn Reson Imaging 38: 288–298. 23737247 10.1002/jmri.23931 PMC3749241

[38] Scheck SM, Boyd RN, Rose SE (2012) New insights into the pathology of white matter tracts in cerebral palsy from diffusion magnetic resonance imaging: a systematic review. Dev Med Child Neurol 54: 684–696. 22646844 10.1111/j.1469-8749.2012.04332.x

[39] Yoshida S, Hayakawa K, Yamamoto A, Okano S, Kanda T, et al (2010) Quantitative diffusion tensor tractography of the motor and sensory tract in children with cerebral palsy. Dev Med Child Neurol 52: 935–940. 20412261 10.1111/j.1469-8749.2010.03669.x

[40] Hoon AH Jr, Stashinko EE, Nagae LM, Lin DD, Keller J, et al (2009) Sensory and motor deficits in children with cerebral palsy born preterm correlate with diffusion tensor imaging abnormalities in thalamocortical pathways. Dev Med Child Neurol 51: 697–704. 19416315 10.1111/j.1469-8749.2009.03306.x PMC2908264

